# Outcome after vacuum-assisted closure in patients with anorectal malformations—a multi-center comparative study

**DOI:** 10.1007/s00423-026-04334-6

**Published:** 2026-10-16

**Authors:** L.-M. Borsch, M. Bouzidi, B. Ludwikowski, M. Bottländer, L. Wennemann, J. Dingemann, N. Schukfeh

**Affiliations:** 1 Department of Pediatric and Adolescent Surgery, Hannover Medical School, 30625 Hannover, Germany; 2 Department of Pediatric Surgery, Children’s and Youth Hospital AUF DER BULT, 30173 Hannover, Germany; 3 Department of Surgery, Division of Pediatric Surgery, Washington University in St. Louis, St. Louis, MO USA

**Keywords:** Congenital abnormalities, Anal Atresia, Anorectal Malformations, Posterior sagittal anorectoplasty, Wound dehiscence, Vacuum-assisted closure, Patient Outcome Assessment

## Abstract

**Purpose:**

In patients with anorectal malformations (ARM), perineal wound complications following posterior sagittal anorectoplasty (PSARP) remain a challenge with an occurrence of 22%-43%. In adults, vacuum-assisted closure (VAC) aims to support wound healing after colorectal procedures. In children evidence is limited, suggesting a potential benefit of VAC therapy after PSARP.

We aimed to evaluate the impact of VAC therapy on outcomes after PSARP in a multicenter comparative study.

**Methods:**

All consecutive patients who underwent PSARP from 01/2018–12/2025 in two participating pediatric surgical departments were included. Complications including postoperative perineal wound dehiscence were analyzed. The study was approved by the local ethics committee (approval number: 12354-BO-K-2026).

**Results:**

48 patients were included: 26 with and 22 without VAC therapy. Baseline characteristics, follow-up duration and the interval until first follow-up were comparable between cohorts respectively. Postoperative outcomes did not differ significantly between groups. Stool smearing occurred in none of the patients in the VAC cohort and in one patient in the non-VAC cohort (p > 0.99). No incontinence was observed in either cohort (p > 0.99). Perineal wound dehiscence occurred in two patients (8%) in the VAC and in one (5%) in the non-VAC cohort (p > 0.99).

**Conclusion:**

In our study, VAC therapy was associated with comparable rates of postoperative wound dehiscence after PSARP compared to the non-VAC cohort and to previous reports. Larger, prospective studies are required to confirm our findings.

## Introduction

Anorectal malformations (ARM) represent a heterogeneous group of congenital anomalies with an incidence of approximately 3.1–3.5 per 10,000 births [1–3] and occur with a slightly higher prevalence in males than in females [1, 4]. Surgical reconstruction has evolved over the past decades compared to earlier abdominoperineal approaches without direct visualization of the sphincter complex, particularly since the introduction of posterior sagittal anorectoplasty (PSARP), which remains the standard procedure for definitive repair in many ARM types [5–7]. Despite these advances in surgical techniques and perioperative management, postoperative complications remain a relevant clinical challenge in this patient population. Among these, perineal wound complications such as wound dehiscence, infection, and impaired healing are of particular concern, as they may prolong hospital stay, delay postoperative recovery, and potentially affect long-term functional outcomes [8–10]. According to the literature, perineal wound complications after PSARP affect 22–43% of patients, highlighting their clinical relevance and impact on postoperative management and long-term outcomes [9].

Negative pressure wound therapy, commonly referred to as vacuum-assisted closure (VAC) therapy, has become an established adjunct in the treatment of complex wounds across multiple surgical specialties. By applying controlled negative pressure to the wound bed, VAC therapy promotes granulation tissue formation, reduces tissue edema and bacterial load, and improves local perfusion, thereby supporting the wound healing process [11–13].

In pediatric surgery, VAC therapy is increasingly being used to manage complex wounds, including abdominal wall defects and sternal wound infections [14–16]. However, its use in the perineal region following reconstructive procedures such as PSARP remains technically challenging and has not been widely studied [17]. Data on the use of VAC therapy after PSARP in children are limited, with only one comparative study available [17].

Postoperative management following PSARP is important for determining long-term outcomes in patients with ARM [18]. Structured anal dilatation programs are commonly used following PSARP, although their necessity and benefit remain under discussion [6, 19]. Adequate perineal wound healing is essential for the successful implementation of these programs and may therefore influence long-term outcomes [19].

Given the limited evidence regarding the use of VAC therapy after PSARP in children with anorectal malformations, further comparative data are required to clarify its potential role in postoperative management. In particular, it remains unclear whether VAC therapy influences postoperative wound complications [17]. Our aim was to evaluate the use of VAC therapy after PSARP and to compare postoperative wound dehiscence and short-term outcomes between patients treated with VAC therapy and a comparable cohort managed without VAC therapy at a second pediatric surgical center.

## Methods

### Ethical approval

This retrospective study was approved by the local ethics committee of Hannover Medical School (approval number: 12354-BO-K-2026).

### Study design and patient cohorts

In this retrospective multicenter comparative study, we included all consecutive patients with anorectal malformation who underwent posterior sagittal anorectoplasty (PSARP) between January 2018 and December 2025 at two tertiary pediatric surgical centers, Hannover Medical School and the Children’s Hospital auf der Bult Hannover. All patients treated at Hannover Medical School received postoperative perineal vacuum-assisted closure (VAC) therapy following PSARP, whereas all patients treated at the Children’s Hospital auf der Bult underwent PSARP without VAC therapy. PSARP was performed using the conventional posterior sagittal approach in all patients with recto-perineal fistula, recto-urethral fistula (bulbar or bladder neck), recto-vestibular fistula and imperforate anus without fistula. Patients with recto-bladderneck fistula underwent laparoscopic-assisted PSARP. Patients with severe multimorbidity as well as those with complex anorectal malformations not suitable for comparison within the study cohort, such as cloaca, Currarino syndrome or rectal atresia, were excluded from the analysis.

Anorectal malformations were classified according to the Krickenbeck classification [20]. The malformation types observed in this study included recto-perineal fistula, recto-urethral fistula (bulbar or bladder neck), recto-vestibular fistula and imperforate anus without fistula. Associated anomalies were assessed systematically. In particular, the presence of VACTERL spectrum anomalies was recorded, and congenital anomalies were categorized according to organ system, including vertebral, anorectal, cardiac, tracheal, esophageal, renal, and limb anomalies. In addition, trisomy 21 was documented.

### Data collection

Demographic and clinical data were retrospectively obtained from electronic medical records. Patient demographics included date of birth, sex, birth weight, age at PSARP, body weight at the time of PSARP, and length of hospital stay.

### Perioperative management and surgical details

Preoperative bowel preparation differed according to institutional protocols. Patients without a protective colostomy received oral bowel preparation. In patients with a protective colostomy, bowel cleansing was performed by antegrade irrigation through the colostomy at Hannover Medical School, whereas at the second center no bowel preparation was applied in these patients. Perioperative data included operating time.

**Fig. 1:**
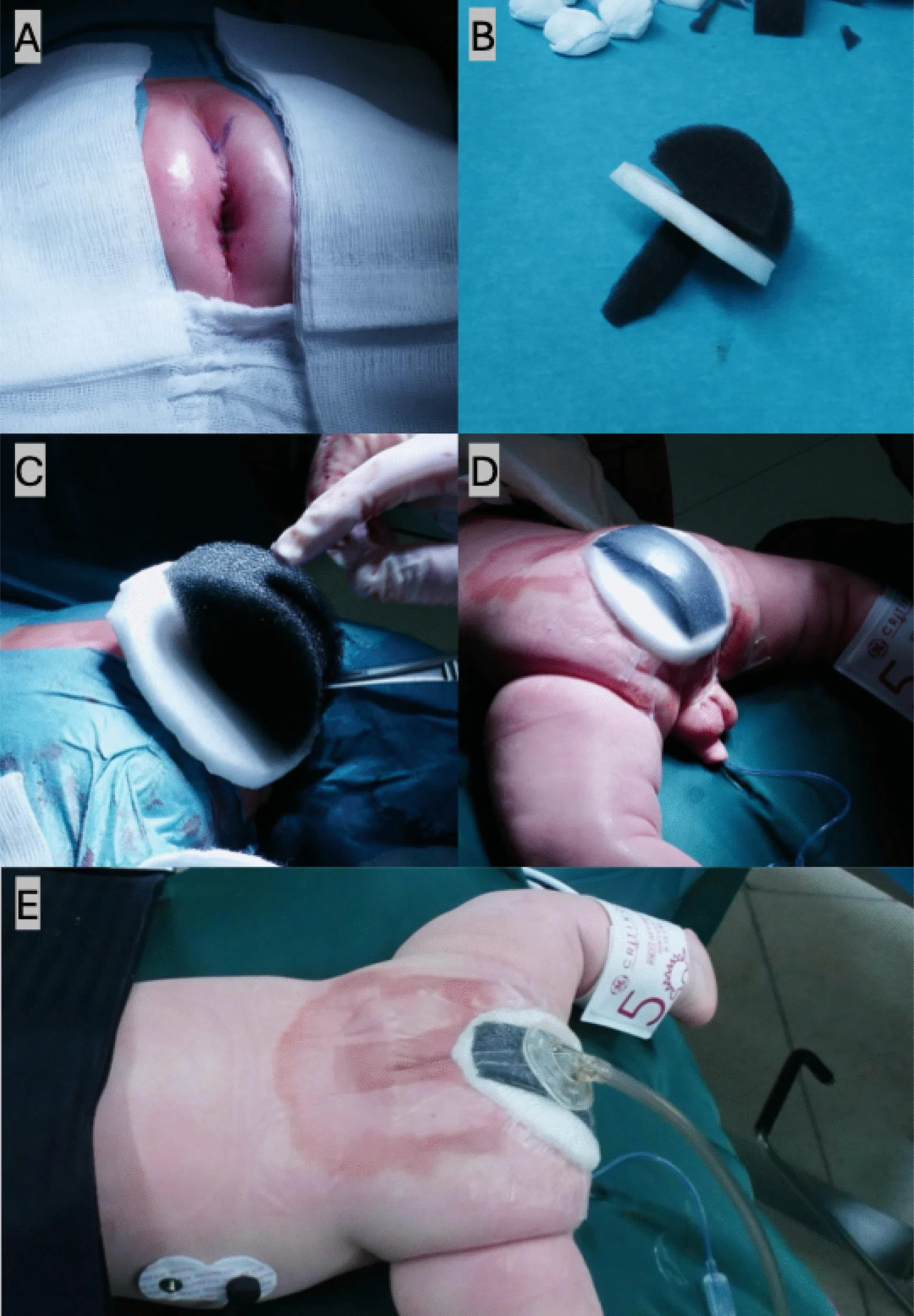
Application of the VAC dressing in a male patient. Neoanus and reconstructed perineum after surgery (**A**). VAC sponge preparation showing trimming of the foam material and interlocking of the components to form a stable and anatomically adapted dressing (**B**). Placement of the VAC dressing in the perineal region (**C**). VAC dressing after complete sealing with adhesive film, before initiation of negative pressure therapy (**D**). VAC dressing after connection to the negative pressure device, demonstrating the completed system (**E**)

In the VAC cohort, endorectal negative pressure wound therapy was applied immediately after completion of the PSARP procedure. A polyurethane foam dressing was positioned in the perineal wound with extension into the neoanal canal and connected to a vacuum device. The technique of VAC application, including placement of the foam within the neoanus and sealing of the perineal region, is illustrated in the intraoperative images (Fig. 1). Continuous negative pressure of 75 mmHg was applied. VAC therapy was typically maintained for 3 days postoperatively, unless earlier removal was required due to loss of seal or leakage.

### Postoperative course

The postoperative course was assessed using several clinical parameters, including time to first stool passage after PSARP (days), time to initiation of enteral feeding (days), duration of total parenteral nutrition (TPN), type and duration of antibiotic therapy. Postoperative management, including timing of enteral feeding and bowel function monitoring, followed institutional protocols.

At Hannover Medical School (MHH), the institutional postoperative protocol included application of VAC therapy for approximately 3 days postoperatively. During this period, patients were kept nil per os. A transurethral urinary catheter was maintained for 3 to 7 days. Antibiotic therapy was continued for the duration of transurethral urinary catheterization. Rectal dilatation was typically initiated 14 days postoperatively and performed by the parents until the target size was reached. Size and frequency were adjusted as needed.

At the second center Children’s Hospital auf der Bult Hannover, postoperative management differed from the protocol at Hannover Medical School. Patients recieved enteral feeding immediately after surgery. Intravenous antibiotic therapy was administered for 5 days. A urinary catheter was maintained for 3 days. Rectal dilatation was initiated according to a protocol according to that used at Hannover Medical School.

### Postoperative complications

Postoperative complications were recorded with particular focus on perineal wound complications. Wound dehiscence of the perineal incision or neoanus was defined as partial or complete separation of the wound edges requiring surgical revision. Complications were classified according to the Clavien–Madadi classification, a pediatric adaption of the Clavien-Dindo system [20, 21]. In addition, the occurrence of revision at surgery was documented, including the time interval between PSARP and revision surgery.

### Follow-up and functional outcomes

Follow-up data were obtained from outpatient records. The time interval between surgery and the first postoperative follow-up visit was recorded. Anal dilatation parameters were documented, including the Hegar dilator size achieved at the first follow-up examination and the final Hegar size after completion of the postoperative dilatation program. The duration of the dilatation program was also recorded. Short-term functional outcomes were assessed based on fecal continence according to Peña, including voluntary bowel movements, soiling and the need for laxative therapy. Fecal incontinence was defined as the involuntary loss of stool, indicating impaired bowel control [6]. Diaper status at the latest available follow-up was additionally recorded to assess whether reliable evaluation of bowel continence was possible.

### Statistical analysis

All data were recorded in Microsoft Excel (Version 16.106 (26,020,821)) and subsequently analyzed using GraphPad Prism (Version 11.0.0 (93), February 10, 2026). Figures were created using Microsoft PowerPoint (Version 16.107 (26,030,819)). Continuous variables are presented as medians with interquartile ranges (IQR), while categorical variables are reported as absolute numbers and percentages. Comparisons between the VAC and non-VAC cohorts were performed using the Mann–Whitney U test for continuous variables and Fisher’s exact test for categorical variables. A p-value of < 0.05 was considered statistically significant.

## Results

**Fig. 2:**
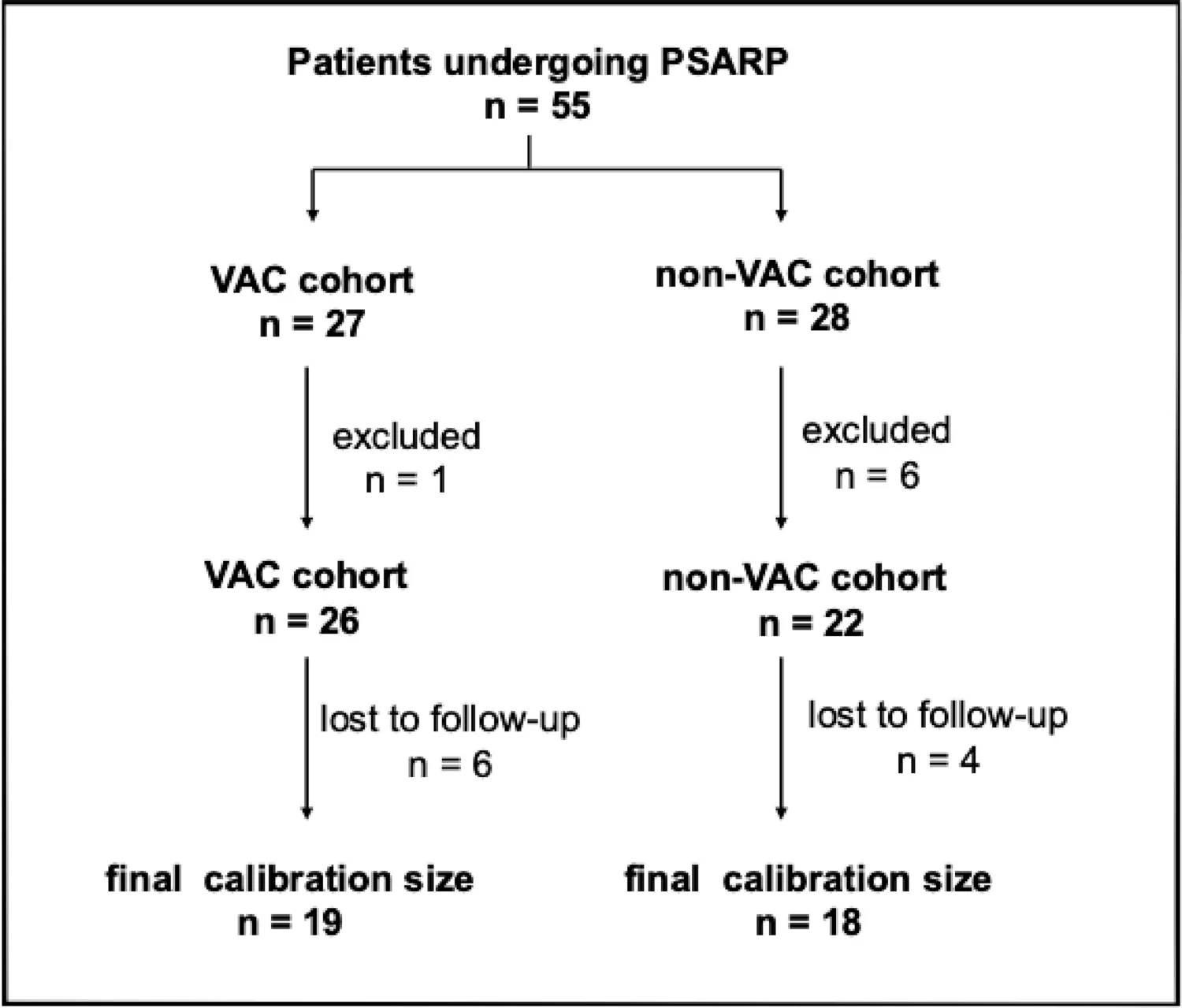
Study flow diagram of patient inclusion and follow-up. Flow diagram of patient selection and follow-up. Fifty-five patients undergoing PSARP were initially identified. Seven patients were excluded due to severe multimorbidity (n = 1), cloaca (n = 4), Currarino syndrome (n = 1), and rectal atresia (n = 1), resulting in a final study population of 48 patients (26 VAC cohort and 22 non-VAC cohort). Follow-up data for the final calibration size were available in 19 VAC patients and 18 non-VAC patients

A total of 55 patients with anorectal malformations who underwent posterior sagittal anorectoplasty (PSARP) during the study period were identified. Seven patients were excluded from the analysis: one patient in the VAC cohort due to severe multimorbidity and six patients in the non-VAC cohort due to cloaca (n = 4), Currarino syndrome (n = 1) and rectal atresia (n = 1). The final study population comprised 48 patients (31 male, 64.6%), including 26 patients in the VAC cohort and 22 patients in the non-VAC cohort. Follow-up data regarding the final achieved calibration size after completion of the dilatation program were available for 19 patients in the VAC cohort and 18 patients in the non-VAC cohort (Fig. 2).

Baseline patient characteristics are summarized in Table 1. A colostomy prior to PSARP was present in 10 patients (38.5%) in the VAC cohort and in seven patients (31.8%) in the non-VAC cohort (p = 0.76). The median age at colostomy was similar between groups (1 day [IQR 1–1.25] vs. 1 day [IQR 1–3], p = 0.8).

**Table 1:** Patient’s demographics. Data are presented as median (IQR) for continuous variables and number (%) for categorical variables. Differences between groups were analyzed using the Mann–Whitney U test for continuous variables and Fisher’s exact test for categorical variables

Patient’s characteristics	VAC cohort (n = 26)	non-VAC cohort (n = 22)	p-value
Demographics			
Sex (male), n (%)	15 (57.7)	16 (72.7)	p = 0.37
Age at PSARP, days	111.5 [IQR 88.8–230.3]	55.5 [IQR 3–119.8]	p = 0.01
Body weight at PSARP, g	5785 [IQR 4983–6875]	5500 [IQR 3685–6555]	p = 0.3
Colostomy, n (%)	10 (38.5)	7 (31.8)	p = 0.76
Age at colostomy, days	1 [IQR 1–1.25]	1 [IQR 1–3]	p = 0.8
Length of hospital stay, days	8 [IQR 7–9]	9 [IQR 5.8–10.8]	p = 0.44
ARM Type			
Rectoperineal fistula, n (%)	14 (53.9) [9 Male, 64.3%]	14 (63.6) [11 Male, 78.6%]	
Recto-urethral-bulbar, n (%)	3 (11.5)	2 (9.1)	
Recto-bladderneck, n (%)	2 (7.7) [2 Male]	2 (9.1) [2 Male]	
Recto-vestibular, n (%)	3 (11.5)	2 (9.1)	
No fistula, n (%)	4 (15.4) [1 Male, 25%]	2 (9.1) [1 Male, 50%]	
Associated malformations			
Vertebral, n (%)	3 (11.5)	7 (31.8)	
Anorectal, n (%)	9 (34.6)	1 (4.5)	
Cardiac, n (%)	13 (50)	6 (27.3)	
Tracheal, n (%)	1 (3.9)	0 (0)	
Easophageal, n (%)	4 (15.4)	2 (9.1)	
Renal, n (%)	7 (26.9)	3 (13.6)	
Limbs, n (%)	5 (19.2)	2 (9.1)	
Syndromes			
VACTERL association, n (%)	8 (30.8)	4 (18.2)	
Trisomy 21, n (%)	2 (7.7)	1 (4.5)	

Regarding ARM types, rectoperineal fistula was the most common malformation in both cohorts, observed in 14 patients in each group (53.9% vs. 63.6%). Other ARM types included rectourethral bulbar fistula (11.5% vs. 9.1%), rectourethral bladder neck fistula (7.7% vs. 9.1%), and rectovestibular fistula (11.5% vs. 9.1%). Cases without fistula were observed in four patients (15.4%) in the VAC cohort and in two patients (9.1%) in the non-VAC cohort.

Associated malformations were frequently observed in both groups. Cardiac anomalies were the most common, occurring in 13 patients (50.0%) in the VAC cohort and in six patients (27.3%) in the non-VAC cohort. Renal anomalies were present in seven patients (26.9%) and three patients (13.6%), respectively, while vertebral anomalies occurred in three patients (11.5%) in the VAC cohort and in seven patients (31.8%) in the non-VAC cohort. A VACTERL association was identified in eight patients (30.8%) in the VAC cohort and in four patients (18.2%) in the non-VAC cohort. Trisomy 21 was present in two patients in the VAC cohort (7.7%) and in one patient in the non-VAC cohort (4.5%).

### Perioperative management and surgical details

At both participating centers, surgical procedures were performed by multiple surgeons. Operating time was 138.5 min (median, IQR 82.3–193.8) in the VAC cohort and 99 min (median, IQR 76–160.5) in the non-VAC cohort (p = 0.12). VAC therapy was applied in all patients in the VAC cohort. In the VAC cohort (n = 26), the median duration of VAC therapy was 3 days (IQR 2–3), and the applied negative pressure was consistently 75 mmHg (IQR 75–75).

### Postoperative course

Postoperative recovery differed between groups due to differences in protocols between the two participating centers (Table 2). Time to first stool passage was significantly longer in the VAC cohort compared to the non-VAC cohort, reflecting differences in postoperative protocols between the two participating centers. Similarly, initiation of enteral feeding occurred later in the VAC cohort. In the VAC cohort, patients with a protective colostomy were started on oral feeding immediately postoperatively. In the non-VAC cohort, oral feeding was initiated immediately in all patients except four. All patients in both cohorts received antibiotic therapy with ampicillin/sulbactam, and no differences in antibiotic regimen were observed between groups.

**Table 2:** Postoperative course. Data are presented as median (IQR) or number (%). Continuous variables were compared using the Mann–Whitney U test and categorical variables using Fisher’s exact test

Parameter	VAC cohort	non-VAC cohort	p-value
Time to first stool passage, days (n = 16/14)	3 (IQR 2–4)	1 (IQR 0–1)	p = 0.0015
Time to enteral feeding, days (n = 26/22)	3 (IQR 0–5)	0 (IQR 0–1)	p = 0.005
Intravenous antibiotic therapy, n (%)	26 (100)	22 (100)	p = 1
Duration of antibiotic therapy, days (n = 24/22)	5 (IQR 3–7)	5 (IQR 3–7)	p = 0.82

### Intraoperative complications

Intraoperative complications occurred in three patients (11.5%) in the VAC cohort. Urethral injuries were observed in two patients, one with a recto-bladder neck fistula and one with an imperforate anus without fistula; in one case, the lesion required primary repair with sutures. In both cases, a transurethral urinary catheter was left in place postoperatively for urinary diversion. In addition, one patient with an imperforate anus without fistula sustained a rectal stump perforation, which was closed intraoperatively using two 5–0 Vicryl sutures. In the non-VAC cohort no intraoperative complications were observed.

### Postoperative complications

Perineal wound dehiscence occurred in two patients (7.1%) in the VAC cohort and in one patient (4.6%) in the non-VAC cohort, without a significant difference between groups (p = 1.00). According to the Clavien–Madadi Classification [21], the majority of patients in both cohorts had minor complications (Grade I B), observed in three patients in the VAC cohort (11.5%) and in five patients in the non-VAC cohort (22.7%). Minor postoperative local erythema or superficial inflammation was observed in three patients in the VAC cohort and four patients in the non-VAC cohort. These findings were managed with local antiseptic wound care and cleansing with sterile compresses and were not classified as wound dehiscence. In the non-VAC cohort, one patient with a recto-urethral bulbar fistula developed postoperative voiding dysfunction following PSARP. Complications requiring pharmacological treatment (Grade II) occurred in two patients in the VAC cohort (7.7%). One patient developed electrolyte imbalance due to Kabuki syndrome with a multicystic dysplastic left kidney and right renal hypoplasia. Additionally, the second patient developed catheter-related sepsis with detection of Staphylococcus hominis five days postoperatively, requiring escalation of antibiotic therapy.

Complications requiring surgical intervention included one patient (3.9%) in the VAC cohort who underwent examination under general anesthesia (Grade IIIa). In addition, one patient (3.9%) in the VAC cohort and one patient (4.6%) in the non-VAC cohort required stoma creation (Grade IIIb). No severe complications requiring intensive care (Grade IV) or postoperative mortality (Grade V) were observed in either cohort.

As shown in Table 3, in the VAC cohort, two patients developed postoperative wound dehiscence. Both patients had a recto-perineal fistula. One patient was male without any associated anomalies or relevant comorbidities. Perineal wound dehiscence occurred on postoperative day 4. The second patient was a female former hypotrophic preterm infant (36 + 4 weeks of gestation, birth weight 1945 g, < 3rd percentile) with multiple associated anomalies, including an aberrant right subclavian artery (arteria lusoria dextra), agenesis of the ductus venosus, a history of right-sided diaphragmatic paralysis with elevation of the diaphragm, tracheobronchomalacia, and severe anemia. This patient fulfilled criteria for a VACTERL association and required creation of a protective stoma due to a complete perineal wound dehiscence on postoperative day 20.

**Table 3:** Complications according to Clavien-Madadi Classification. Data are presented as number (%). Differences between groups were analyzed using Fisher’s exact test

Variable	VAC cohort (n = 26)	non-VAC cohort (n = 22)	p-Value
Wound dehiscence, n (%)	2 (7.1)	1 (4.6)	p = 1
Clavien-Madadi Classification [21]			
(I A) Any deviation from the planned course due to management and/or organizational problems, n (%)	0 (0)	0 (0)	
(I B) Any deviation from the planned *clinical* course, n (%)	3 (11.5)	5 (22.7)	
(II) Medically treated, n (%)	2 (7.7)	0 (0)	
(III A) Examined under general anaesthesia, n (%)	1 (3.9)	0 (0)	
(III B) Stoma establishment, n (%)	1 (3.9)	1 (4.6)	
(IV) Intensive care, n (%)	0 (0)	0 (0)	
(V) Death, n (%)	0 (0)	0 (0)	

In the non-VAC cohort, one patient developed postoperative wound dehiscence requiring revision surgery. The patient was a female with a rectovestibular fistula and Pallister–Killian syndrome associated with vertebral anomalies. Wound dehiscence occurred on postoperative day 5, and surgical revision was performed on postoperative day 13, including creation of a descending colostomy and application of VAC therapy afterwards.

### Follow-up and functional outcomes

The interval between surgery and the first follow-up examination did not differ significantly between cohorts, with a median of 18 days (IQR 14–20) in the VAC cohort and 16.5 days (IQR 14.8–21.5) in the non-VAC cohort (p = 0.75). At the first follow-up examination, the Hegar size was significantly larger in the VAC cohort compared to the non-VAC cohort (n = 21/21, median 10 mm [IQR 10–11.8] vs. 8.5 mm [IQR 8–9.5], p < 0.0001). Similarly, the final achieved Hegar size after completion of the dilatation program was greater in the VAC cohort (n = 19, median 14 mm [IQR 13–15]) than in the non-VAC cohort (n = 18, median 12.3 mm [IQR 12–13], p < 0.0001). The duration of the dilatation program did not differ significantly between groups (n = 17/14, median 7 months [IQR 5.5–11] vs. 9 year [IQR 3.75–11.75], p = 0.45). The median follow-up duration was 20 months (IQR 8–35) in the VAC cohort and 17 months (IQR 11–48.5) in the non-VAC cohort, without a significant difference between groups (p = 0.87). At the latest follow-up, 18/21 patients (85.7%) in the VAC cohort and 15/18 patients (83.3%) in the non-VAC cohort were still wearing diapers (p > 0.99). Regarding functional outcomes, stool smearing occurred in none of the patients in the VAC cohort and in one of the none- VAC patients (p = 0.45). Reliable assessment of bowel incontinence was possible in 18 patients (85.7%) in the VAC cohort and 15 patients (83.3%) in the non-VAC cohort, who did wear diapers at the time of the follow-up examination. In these patients, no cases of fecal incontinence were documented in either cohort (p > 0.99). The use of laxatives after PSARP was comparable between groups (11 patients [42.3%] in the VAC cohort vs. 13 patients [59.1%] in the non-VAC cohort, p = 0.39). Table 4

**Table 4:** Follow-up and functional outcomes. Data are presented as median (IQR) for continuous variables and number (%) for categorical variables. Continuous variables were compared using the Mann–Whitney U test and categorical variables using Fisher’s exact test. The number of patients included in each analysis is indicated for each variable

Outcome	VAC cohort	non-VAC cohort	p-value
First appointment after surgery, days (n = 26/22)	18 [IQR 14–20]	16.5 [IQR 14.8–21.5]	p = 0.75
Hegar size at first appointment (mm), (n = 21/21)	10 [IQR 10–11.8]	8.5 [IQR 8–9.5]	p < 0.0001
Hegar size after dilatation program (mm), (n = 19/18)	14 [IQR 13–15]	12.3 [IQR 12–13]	p < 0.0001
Duration of dilatation program (months), (n = 17/14)	7 [IQR 5.5–11]	9 [3.75–11.75]	p = 0.89
Follow-up duration, months (n = 19/21)	20 [IQR 8–35]	17 [IQR 11–48.5]	p = 0.87
Diaper use at latest follow-up, n (%), (n = 24/21)	18 (85.7%)	15 (83.3%)	p > 0.99
Stool smear, n (%), (n = 26/21)	0 (0%)	1 (4.6%)	p = 0.45
Incontinence, n (%), (n = 26/22)	0 (0%)	0 (0%)	p > 0.99
Use of laxatives after PSARP, n (%), (n = 26/26)	11 (42.3%)	13 (59.1%)	p = 0.39

### Long-term complications

Eight months after PSARP, one patient from the VAC cohort developed recurrent obstructive symptoms consistent with mechanical ileus, including vomiting and absence of stool passage despite conservative management. Exploratory laparotomy showed dilated small bowel loops without a clear mechanical obstruction, leading to ileostomy creation. The further course was uneventful, with no recurrence of constipation, no need for laxatives, and no stool smearing. In the non-VAC cohort no long-term complications were observed.

## Discussion

Perineal wound dehiscence is a common complication following PSARP, with reported rates ranging from 22 to 43% according to the literature [9]. Previous studies have suggested that the occurrence of wound dehiscence is influenced by factors such as malformation type, perioperative management, and the use of a protective colostomy, although consistent independent risk factors have not been clearly identified [8, 9, 22].

In comparison, our study demonstrated low rates of wound dehiscence in both cohorts (7.1% vs. 4.6%), with two cases in the VAC cohort and one case observed in the non-VAC cohort, without a statistically significant difference between both cohorts (p = 1). Wound dehiscence occurred in both cohorts. As expected, no wound dehiscence was observed in patients with a protective colostomy, supporting the protective role of fecal diversion on perineal wound healing [5]. These findings suggest that the relationship between fecal diversion and wound healing may be more complex and influenced by patient-specific factors. Our findings do not indicate a relevant impact of VAC therapy on this outcome.

VAC therapy promotes granulation tissue formation, reduces edema, and improves local perfusion, thereby supporting wound healing, particularly in the perineal region, where contamination and mechanical stress may impair healing [11–13]. In adult colorectal surgery, VAC therapy has also been increasingly applied to the perineal wound following major abdominoperineal procedures, particularly after abdominoperineal resection (APR) or extralevator abdominoperineal excision (ELAPE) for rectal cancer. These types of surgery are associated with a high incidence of perineal wound complications and VAC therapy has been investigated as a strategy to reduce wound-related morbidity and to support perineal wound healing in this context [23, 24].

Evidence on the use of VAC therapy after PSARP remains limited, with only one comparative study by Martynov et al. evaluating its application in children with anorectal malformations in comparative study across two centers. The authors reported a lower incidence of perineal wound dehiscence in patients treated with VAC therapy compared with conventional postoperative management, with no cases observed in the VAC cohort compared to rates of up to 25% in a historical control cohort, suggesting a potential benefit of VAC therapy in supporting perineal wound healing. However, the study focused primarily on short-term postoperative outcomes [17].

In contrast, our study demonstrated low rates of wound dehiscence in both cohorts (7.1% vs. 4.6%), without evidence of a statistically significant difference between VAC and non-VAC therapy. This discrepancy may be explained by several differences. First, Martynov et al. used a historical control group, whereas our study compared simultaneous cohorts, which may reduce the influence of temporal bias related to changes in surgical and perioperative practices. Second, their study was conducted across two centers, both of which applied VAC therapy, where surgical techniques and postoperative management may have been more consistent, whereas our multicenter design includes different centers with different postoperative protocols and potentially varying surgical approaches, which may have introduced variability in treatment. Third, differences in patient characteristics and malformation complexity may have influenced the observed outcomes. Martynov et al. included only patients with rectoperineal and rectovestibular fistulas, whereas our study included a broader spectrum of anorectal malformations according to the Krickenbeck classification [20]. In addition, it remains unclear whether the reported benefit is attributable to VAC therapy alone or to differences in perioperative management, including feeding strategies and postoperative care in both studies.

Despite these differences, both studies showed low rates of wound dehiscence in patients treated with VAC therapy. However, the similarly low rates in both groups in our study suggest that VAC therapy alone may not explain these results. Notably, the overall incidence of wound dehiscence in our study was substantially lower than the rates reported in the literature following PSARP, which range from 22 to 43% [9]. This discrepancy may reflect differences in patient selection, surgical technique and perioperative management. In contrast to adult colorectal surgery, where VAC therapy has been applied in high-risk perineal wounds and may contribute to improved healing [23, 24], the baseline risk of wound dehiscence in our pediatric cohort appears comparatively low. Therefore, the additional benefit of VAC therapy in this setting may be limited and potentially overshadowed by other factors such as surgical technique, perioperative management and underlying malformation type.

Differences in postoperative management, including delayed initiation of enteral feeding and later first stool passage in the VAC cohort, have not been reported in the literature. These observed differences in the early postoperative course in the VAC cohort are more likely explained by differences in postoperative management protocols between the participating pediatric surgical centers rather than by the VAC therapy itself. In particular, the application of VAC therapy in the perineal region influences postoperative management, leading to a more cautious feeding strategy to limit wound contamination. Standardized postoperative protocols following PSARP have been associated with improved outcomes and reduced complication rates [10, 18]. As VAC therapy is not part of standardized postoperative care, center-specific differences in postoperative management may have influenced the results and should be considered when interpreting these findings.

Following PSARP, functional outcomes are known to be influenced by multiple factors, including malformation type, associated anomalies, and surgical reconstruction [18, 25]. Functional outcomes in patients with anorectal malformations may not be fully apparent in early childhood and often become more evident at older ages, as continence has been shown to improve over time following PSARP [26, 27]. In our study short-term functional outcomes were comparable between the VAC and non-VAC cohort. No cases of fecal incontinence were observed and stool smearing occurred in one patient in the non-VAC cohort. However, 18 VAC patients (85.7%) and 15 non-VAC patients (83.3%) were still wearing diapers at the latest follow-up, limiting the reliable assessment of bowel continence. Therefore, both the young age of the patients and the limited follow-up duration restrict the interpretation of long-term functional outcomes.

The clinical relevance of postoperative anal dilatation parameters remains uncertain, and current evidence suggests that long-term functional outcomes are not only determined by achieved dilator size [19]. In our study, patients in the VAC cohort achieved significantly larger Hegar dilator sizes during follow-up. However, this finding is likely influenced by differences in dilatation protocols and center-specific practices rather than a direct effect of VAC therapy.

This study has limitations. The retrospective design is associated with a potential risk of selection bias and limited control over confounding variables. The relatively small sample size limits statistical power. Furthermore, differences in patient characteristics and underlying malformations between the two pediatric surgical centers may have influenced the results. Finally, the incomplete follow-up data in some patients limits the evaluation of long-term functional outcomes.

## Conclusion

VAC therapy following PSARP may represent a useful approach, although low rates of wound dehiscence were observed in both the VAC and non-VAC cohort [17]. In our study no significant difference in wound dehiscence was observed compared to postoperative management without VAC therapy. Postoperative outcomes may be influenced by center-specific protocols and patient-related factors. Randomized controlled trials are required to further define the role of VAC therapy in postoperative management following PSARP.

## Data Availability

No datasets were generated or analysed during the current study.
